# Development of Vancomycin Delivery Systems Based on Autologous 3D Platelet-Rich Fibrin Matrices for Bone Tissue Engineering

**DOI:** 10.3390/biomedicines9070814

**Published:** 2021-07-13

**Authors:** Arita Dubnika, Karina Egle, Marite Skrinda-Melne, Ingus Skadins, Jayakumar Rajadas, Ilze Salma

**Affiliations:** 1 Rudolfs Cimdins Riga Biomaterials Innovations and Development Centre, Institute of General Chemical Engineering, Riga Technical University, LV-1658 Riga, Latvia; karina.egle@rtu.lv (K.E.); marite.skrinda@rtu.lv (M.S.-M.); 2 Baltic Biomaterials Centre of Excellence, Headquarters at Riga Technical University, LV-1658 Riga, Latvia; salma.ilze@rsu.lv; 3 Department of Biology and Microbiology, Rīga Stradiņš University, LV-1007 Riga, Latvia; ingus.skadins@rsu.lv; 4 Department of Medicine and Advanced Drug Delivery and Regenerative Biomaterials Laboratory of Cardiovascular Institute, Stanford University School of Medicine, Palo Alto, CA 94304, USA; jayraja@stanford.edu; 5 Institute of Stomatology, Rīga Stradiņš University, LV-1007 Riga, Latvia

**Keywords:** platelet-rich fibrin, drug delivery, liposomes, microcapsules, vancomycin, phospholipids, PLGA, drug release, microtomography

## Abstract

Autologous platelet-rich fibrin (PRF) is derived from the blood and its use in the bone tissue engineering has emerged as an effective strategy for novel drug and growth factor delivery systems. Studies have approved that combined therapy with PRF ensures higher biological outcomes, but patients still undergo additional treatment with antibiotic drugs before, during, and even after the implantation of biomaterials with PRF. These systematically used drugs spread throughout the blood and lead not only to positive effects but may also induce adverse side effects on healthy tissues. Vancomycin hydrochloride (VANKA) is used to treat severe *Staphylococcal* infections but its absorption in the target tissue after oral administration is low; therefore, in this study, we have developed and analyzed two kinds of VANKA carriers—liposomes and microparticles in 3D PRF matrices. The adjustment, characterization, and analysis of VANKA carriers in 3D PRF scaffolds is carried out in terms of encapsulation efficiency, drug release kinetics and antibacterial activity; furthermore, we have studied the micro- and macrostructure of the scaffolds with microtomography.

## 1. Introduction

The use of platelet-rich fibrin (PRF) in tissue engineering has emerged as an effective strategy for novel drug delivery systems [1]. Autologous PRF is derived from the blood by centrifugation and contains growth factors and cells (leucocytes and platelets). With an adjusted PRF preparation method, it can be stable in liquid phase for up to 30 min before coagulation, thus allowing to prepare a scaffold for the drug delivery system and use it as an injectable system [2,3]. The use of autologous PRF can improve the biological outcomes of the bone and tissue regeneration procedures, especially in maxillofacial surgeries [4,5,6]. Previous clinical studies have approved that for patients receiving therapy combined with PRF, the biological outcomes and healing were better [7,8,9,10].

Systematically used drugs, without a specific carrier, spread throughout the body and often drug degradation rate is relatively short. It ensures not only a positive effect on the damaged tissue, but may also induce adverse side effects on healthy tissues [11]. The aim of drug delivery systems is to achieve the highest therapeutic effect with the lowest drug concentration [12]. Vancomycin hydrochloride (VANKA) is a water-soluble tricyclic glycopeptide antibiotic [13] that prevents/destroys several Gram-positive microorganisms, which are the most common pathogens. It is used to treat severe *Staphylococcal* infections, in particular methicillin-resistant *S. aureus* (MRSA) and is included in anti-cancer medicines. VANKA is applied in cases when penicillin is ineffective or causes allergic reactions, as well as for the treatment of infections where other antibiotics are resistant [14,15]. VANKA is administered intravenously by injections or infusions, as well as perorally in the form of capsules. A lack of oral administration explains the low VANKA absorption. Within 24 h, 80 to 90% of the orally administered VANKA is excreted unchanged in the urine. The average duration of VANKA therapy is 7 to 10 days [16]. According to clinical studies, VANKA microbiologic inhibitory activity (MIC) to the strains of MRSA ranges from 0.125 μg/mL to 2 μg/mL [17,18,19]. Additionally, over time, VANKA degrades into crystalline degradation products–chiral stationary phase, CDP-1; thus, the concentration of the VANKA active form decreases and, while monitoring the drug concentration, an appropriate method has to be selected [20,21,22].

VANKA can be encapsulated into the liposomes, which are unique medication carriers, because they overcome the disadvantages of the peroral or intravenously administered drugs. The most important liposomal properties are their biocompatibility, the ability to encapsulate a large amount of the substance, the ability to increase the stability of the encapsulated substance, controlled drug delivery and limited circulation time in the human body [23]. The main materials for liposome preparation are phospholipids and cholesterol [24]. Phospholipids are lipids composed of a polar and non-polar part that contain phosphorus and provide a spherical liposome formation process [12]. Cholesterol is added to the liposome composition to reduce the permeability of fluids through the membrane and increase liposome stability as well as promote the increase of phospholipid bilayer viscosity [24,25,26]. Nevertheless, the encapsulation efficiency of VANKA in liposomes depends on the preparation method and lipid composition, but it is usually below 20% [14,27]; higher encapsulation efficiency (33%) could be achieved by freezing/thawing [28]. As an alternative, polymeric microparticles from poly lactic-co-glycolic acid (PLGA) have been studied to encapsulate VANKA [25,27,28]. PLGA is a biodegradable copolymer that has been FDA approved and is used as a drug carrier. Drug release kinetics can be controlled by the polymerization rate of lactide and glycolide as well as molecular weight of PLGA [27,29]. Both liposomes and PLGA microcapsules have drawbacks in terms of encapsulation efficiency, but encapsulation of VANKA in carrier provides high clinical benefits for the long-term use of antibiotics. Therefore, the adjustment of VANKA carrier composition and the drug release rate in autologous samples is essential. Current studies on drug carriers within the autologous PRF samples are limited. Wang et al. studied platelet poor plasma-based fibrin gel containing liposomes/chitosan scaffold for hydrophilic drug delivery. The crosslinking process with glutaraldehyde ensured the drug delivery up to 18 days [30]. Micro- and nanoparticles and fibrin are studied in terms of their interaction to activate coagulation cascade [31], as well as to create double networks/hydrogels consisting of gelatin and PRF for bone healing [32]. Furthermore, PRF compositions are studied to develop three-dimensional networks with calcium phosphate granules [33], collagen membranes [34], and other materials [1], but these strategies are targeted towards delivery of autologous growth factors and cells that are within the PRF.

In this study, we aimed to develop controlled VANKA delivery systems based on PRF scaffold, combining antibacterial properties of VANKA against MRSA with autologous PRF scaffold containing living cells and growth factors.

## 2. Materials and Methods

### 2.1. Materials

VANKA hydrochloride (Sigma-Aldrich, St. Louis, MI, USA), polylactic-co-glycolic acid (PLGA; Resomer RG 502, Evonik Nutrition & Care GmbH, Essen, Germany), cholesterol (≥99%, M = 386.65 g/mol, Sigma-Aldrich, St. Louis, MI, USA), 1,2-distearoyl-sn-glycero-3-phosphocholine (DSPC; >99%, M = 790.145 g/mol, Avanti Polar Lipids, Alabaster, Alabama), chloroform (≥99%, M = 119.38 g/mol, Sigma-Aldrich, St. Louis, MI, USA), acetonitrile (≥99.9%, Sigma-Aldrich, St. Louis, MI, USA), di-potassium hydrogen phosphate trihydrate (K_2_HPO_4_; ≥99%, Merck, Darmstadt, Germany), polyvinyl alcohol (PVA; M = 25 kDa, 88 mol% hydrolysis, Polysciences, Warrington, UK), phosphoric acid (H_3_PO_4_; C = 75% *w*/*w*, Latvijas ķīmija, Riga, Latvia), dichloromethane (DCM; ≥99.8%, Merck, Darmstadt, Germany), methanol (≥99.9%, Sigma-Aldrich, St. Louis, MI, USA), phosphate-buffered saline tablet (PBS, Sigma-Aldrich, St. Louis, MI, USA), Lugol solution (Deltalab Eurotubo^®^, Barcelona, Spain).

### 2.2. Blood Collection and Separation of PRF

Blood of 10 healthy volunteers was collected in 10 mL i-PRF+ tubes and immediately placed in the centrifuge (“PRF Duo Quattro”). A medical nurse drew the blood with a clinically approved butterfly blood collection method (“BC-12, 21 G × 3/4”). The centrifugation time was 3 min and the rotor radius was 100 mm, 700 rpm. After the centrifugation, the upper layer of liquid PRF (1 mL) from each tube was transferred into a 24-well cell culture plate for further use.

Written consent from all of the volunteers for use of their samples in the research studies was obtained. All donors were free of any infectious disease and did not have any abnormal consumption of nicotine or alcohol. None of the subjects used any drugs for anticoagulation. Permission No. 6-2/10/53 of the Research Ethics Committee of Riga Stradins University has been received for the research.

### 2.3. Preparation of VANKA Carriers

#### 2.3.1. Preparation of Liposomes

Liposomes composed of DSPC and cholesterol in a molar ratio of 2:1 were synthesized using a thin film hydration method and three dehydration–rehydration cycles [14,15]. To obtain a thin film, DSPC and cholesterol were dissolved in chloroform. The sample was then dried in a stream of nitrogen gas until dry lipid film formation on the vessel walls was observed. To dry completely, sample was placed at −400 mbar vacuum for 6 h. Hydration was carried out gradually by adding 1 mL of deionized water to the sample and treating the sample in an ultrasonic bath to reduce liposome size and agglomeration. Hydration was complete when 5 mL of deionized water were added to the sample. Hydrated liposomes were frozen and lyophilized. Three dehydration–rehydration cycles were performed to reduce liposome size.

VANKA-containing liposomes were synthesized according to the method described above. The concentration of VANKA in hydration solution was 250 μg/mL. Hydrated sample contained 6.25% VANKA from the total sample mass. During the second and third dehydration–rehydration cycles, VANKA-containing liposome samples were hydrated in 5 mL of deionized water.

#### 2.3.2. Preparation of Microcapsules

For the preparation of microcapsules, the water–oil–water method was used. VANKA was dissolved in deionized water (10 mg/mL). This solution was added to the organic phase solution that consists of a PLGA polymer (1 g) dissolved in DCM (5 mL). Both solutions were stirred vigorously to yield a water-in-oil emulsion. Afterwards, the water-in-oil primary emulsion was added to 100 mL PVA aqueous solution (4 wt %) and further mixing was carried out for 30 to 60 s at a rate of 600 rpm [28]. The resulting emulsion was transferred to 2.5 L deionized water and stirred for 1 h. The suspension was centrifuged and frozen in liquid N_2_, then lyophilized for 72 h to obtain a dry microcapsule powder.

### 2.4. Incorporation of VANKA Carriers in PRF

The VANKA carriers were added to the PRF by suspending them in PRF with an automatic pipette before the clot formed. As control samples, PRF without VANKA carriers were prepared using the same procedure. The mixing procedures were carried out in sterile 24-well cell culture plates.

Nine different samples with corresponding abbreviations were prepared—see Table 1 below:

### 2.5. Characterization of VANKA Carriers and PRF Scaffold

#### 2.5.1. Drug Load and Encapsulation Efficiency

The drug load (DL) in liposomes and VANKA encapsulation efficiency was determined indirectly by measuring free VANKA in the liposome suspension. The sample preparation included liposome suspension centrifugation at 3000 rpm for 15 min (“Biosan LMC-3000”). To prepare the sample for VANKA determination, 1 mL of solution was filtered through 0.22 µm nylon membrane filter and then analyzed using ultra-performance liquid chromatography (see Section 2.6).

VANKA encapsulation efficiency was calculated using the equation below [13].

(1) $${\mathrm{EE}}_{L}=\frac{W_{\mathrm{total}}-{\;W}_{\mathrm{free}}}{W_{\mathrm{total}}}\cdot 100\%,$$

where W_total_ is the amount of drug (VANKA) added in liposomes and W_free_ is the detected amount of VANKA in the solution. The drug load (%) in liposomes was calculated according to Equation (2), where w_lipid_ is the amount of lipids added in the system:

(2) $${\mathrm{DL}}_{L}=\frac{{\;W}_{\mathrm{total}}-W_{\mathrm{free}}}{W_{\mathrm{lipid}}}\cdot 100\%,$$

To determine the total drug load (DL) in microcapsules, nitrogen content microanalysis (apparatus—Vario MACRO CHNS, Hanau, Germany) was used. Calculations were carried out according to Equation (3):

(3) $${\mathrm{DL}}_{\mathrm{MK}}=\frac{N_{\mathrm{el}}}{N_{\mathrm{tot}}}\cdot 100\%,$$

where N_el_ is the nitrogen content found using microanalysis and N_tot_ is the calculated nitrogen content in VANKA. The encapsulation efficiency (EE) was calculated according to Equation (4):

(4) $${\mathrm{EE}}_{\mathrm{MK}}=\frac{\mathrm{DL}\cdot w_{\mathrm{microcapsules}}}{W_{\mathrm{total}\mathrm{VANKA}}}\cdot 100\%,$$

where w_microcapsules_ is the number of microcapsules obtained and w_totalVANKA_ is the amount of VANKA added in the microcapsule preparation process.

#### 2.5.2. Particle Size

The average liposome size and particle size distribution was determined using a dynamic light scattering (DLS) analyzer and BIC Particle Sizing Software version 4.03. Measuring temperature 20 °C, angle 90°, 2 min per measurement, refractive index 1.45.

The average microcapsule size and particle size distribution was determined using a laser particle size analyzer (ANALYSETTE 22, measuring range from 0.01 to 1000 μm, laser wavelength 650 nm).

Each sample was measured in triplicate.

#### 2.5.3. Morphology

The morphology of hydrated liposomes and as prepared PRF with and without carriers was determined by light microscope “Leica DFC320, DMLP”. The morphology and size of the negatively stained liposomes was characterized with a transmission electron microscope (TEM) “JEOL JEM1230”. We used SEM/energy dispersive spectroscopy (EDS) for semi-quantitative chemical analysis of the lyophilized liposomes, microcapsules, and PRF samples. The samples were analyzed with an Oxford *X-Max^N^* Silicon Drift Detector (150 mm^2^) attached to Tescan *Mira\LMU* SEM. Five randomly selected areas of at least 100 × 100 μm were analyzed on the surface of each sample at an accelerating voltage of 15 kV, working distance of 27 mm, and a counting time of 60 s.

#### 2.5.4. Chemical Structure and Phase Composition

The obtained carriers and lyophilized PRF chemical structures were determined by Fourier-transform infrared spectroscopy (FTIR). FTIR (“Varian 800 FT-IR”, Scimitar Series, Randolph, MA 02368, USA) spectra were recorded in the attenuated total reflectance (ATR, “GladiATRTM”, Pike Technologies, Madison, WI 53719, USA) mode. Spectra were obtained at 4 cm^−1^ resolution co-adding 50 scans over a range of wavenumbers from 400 cm^−1^ to 4000 cm^−1^. Before every measurement, a background spectrum was taken and deducted from the sample spectrum.

#### 2.5.5. Micro-Computed Tomography Analysis

To visualize 3D PRF samples, high-resolution micro-computed tomography (µCT50, Scanco Medical AG, Bruttisellen, Switzerland) was used. Samples were stained with a common soft tissue staining procedure that increases the contrast in non-mineralized biological tissues [35]. Staining was carried out with 0.3% Lugol’s solution [36] to see the drug delivery systems within the PRF samples. PRF sample with/without drug delivery systems was placed in 0.3% Lugol’s solution for 1 min, then rinsed with water. The following scanning parameters were used: 55 kVp voltage, 109 µA current, 7.4 µm voxel size, 0.1 mm Al filter, frame averaging = 6, and 360° rotation. The scanning time for each sample was 6 h and 40 min. Reconstruction of 3D datasets from microCT projection data, including beam hardening correction, was performed automatically after completion of each cone beam image stack. The visualization module performs sophisticated 3D rendering of large data sets using high-quality ray-tracing algorithms.

### 2.6. Assessment of VANKA Release Kinetics

**Figure 1:**
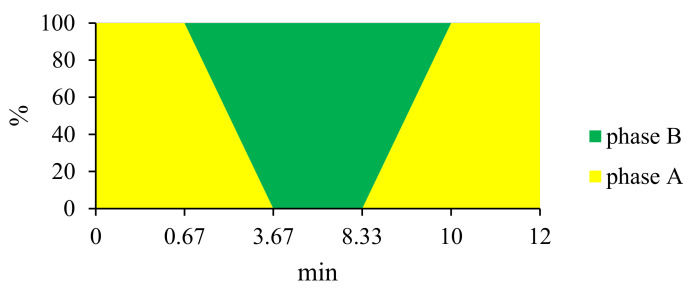
Gradient curve of mobile phase composition for VANKA quantification method.

Assessment of VANKA concentration and release kinetics was analyzed using ultra-performance liquid chromatography. The chromatographic method was transferred to the previously described method on active form VANKA detection on HPLC. “Waters Acquity UPLC H-class” with UV/VIS detector “Waters Acquity TUV” set at 210 nm and the chromatography column “Waters Acquity UPLC BEH C18, 1.7 μm, 2.1 × 50 mm” was used. The mobile phase consisted of two phases: A—0.05 M K_2_HPO_4_ × 3H_2_O buffer (pH = 3.2): acetonitrile: methanol in ratio (*v/v*) 91:5:4, respectively; and B—0.05M K_2_HPO_4_ × 3H_2_O buffer (pH = 3.2): acetonitrile: methanol in ratio (*v/v*) 84:8:8, respectively [22]. Separation of the active VANKA form was obtained using the following gradient (Figure 1) conditions at a flow rate of 0.3 mL/min: 100% A for 0.67 min, change to 100% B in 3.0 min, hold B for 4.66 min, change to 100% A in 1.67 min and hold for 2 min. The total run time for one sample was 12 min. The column temperature was maintained at 30 °C ± 5 °C and sample temperature at 10 °C ± 5 °C.

Samples for the evaluation of in vitro VANKA release from the liposomes were immersed in 10 mL PBS, using dialyze membrane, and incubated at 37 °C ± 0.5 °C and 50 rpm (Environmental Shaker—incubator ES-20, Biosan, Riga, Latvia). A total of 250 µL aliquots of the solution were taken directly from the vessels after 1 h, 2 h, 3 h, 5 h, 24 h, 48 h, 72 h, 96 h, and 168 h. The volume taken was replaced with 250 µL of fresh PBS, keeping the total dissolution medium volume constant.

PLGA_µC_VANKA and PRF/VANKA carriers were immersed in 10 mL deionized water and incubated at 37 °C ± 0.5 °C and 50 rpm (Environmental Shaker—incubator ES-20, Biosan, Riga, Latvia). One milliliter aliquots of the solution were taken directly from the vessels after 1 h, 2 h, 4 h, 6 h, 24 h, 48 h, 72 h, 146 h, 217 h, and 239 h. The volume taken was replaced with 1 mL of deionized water, keeping the total dissolution medium volume constant. The cumulative fraction of the release rate was calculated from the following equation [15]:

(5) $$Release\;rate=\frac{c_{n}v_{0}+\sum_{i=0}^{n-1}c_{i}v_{i}}{c_{0}v_{0}}\cdot 100\%,$$

where *c_n_* is the VANKA concentration in the release medium of each time interval, *v*_0_ is the total volume of the release medium, *v_i_* is the volume of the withdrawn medium, *c_i_* is the drug concentration in the release medium at time, and *c*_0_ is the total VANKA concentration in the system.

### 2.7. Preparation of Bacterial Suspension and Inoculum for Antibacterial Tests

Antibacterial properties were tested via the disk diffusion test, also known as the Kirby-Bauer disk diffusion method, which is a standardized method used in microbiology laboratories in order to determine bacterial susceptibility against antibiotic substances. Bacterial suspension of *Staphylococcus aureus* (*ATCC 25923*) reference culture was prepared according to EUCAST (European Committee on Antimicrobial Susceptibility Testing) standards in optic density of 0.5 according to McFarland standard with McFarland optic densitometer (Biosan, Riga, Latvia). Bacterial suspension was inoculated onto a sterile Mueller–Hinton (MH) agar (Oxoid, Altrincham, UK) with a sterile cotton swab.

### 2.8. Determination of VANKA Loaded PRF Antibacterial Properties

After bacterial inoculation, samples were placed onto MH agar with sterile forceps, and MH agar with samples was incubated in the thermostat for 24 h at 37 °C degrees. After 24 h, the antibacterial properties of the samples were analyzed by measuring the sterile area (diameter) around the samples. After the measurements, a new bacterial suspension was prepared and inoculated onto a new sterile MH agar, and the samples were transferred from the old to the new MH agar and incubated for another 24 h at 37 °C. These actions were repeated every 24 h until no trace of antibacterial characteristics or sterile area around the samples was found for two days in a row.

### 2.9. Determination of Antibacterial Properties of Sample Incubation Medium

The samples were immersed in the medium and incubated at 37 °C ± 0.5 °C and 50 rpm. One milliliter aliquots of the solution were taken directly from the vessels after 1 h, 2 h, 4 h, 18 h, 24 h, 48 h, 70 h, 90 h, 146 h, 217 h, and 239 h. The volume taken was replaced with 1 mL of deionized water, keeping the total dissolution medium volume constant. After bacterial inoculation, the nitrocellulose disks were placed on MH agar and impregnated with 20 μL of incubation solutions. Three nitrocellulose disks from one series were used. MH agar were incubated in the thermostat for 24 h in 37 °C degrees. After 24 h, antibacterial properties were analyzed by measuring the sterile area (diameter) around the disks.

### 2.10. Statistical Evaluation

All results were expressed as the mean value ± standard deviation (SD) of at least three independent samples. The significance of the results was evaluated using an unpaired Student’s *t*-test with the significance level set at *p* < 0.05. One- and two-way analyses of variance (ANOVAs) were performed to evaluate the differences between the results.

## 3. Results

### 3.1. Evaluation of VANKA Carriers

#### 3.1.1. Particle Size Distribution and Morphology

In order to develop a controlled VANKA delivery system based on PRF, VANKA was encapsulated in two carrying systems—liposomes and microcapsules, respectively. Conventional methods for preparation of liposomes and microcapsules were chosen to establish the difference between the VANKA carrying materials within the PRF scaffolds. Nevertheless, VANKA is hydrophilic drug and according to the previous studies, the encapsulation efficiency in both liposomal and microcapsule systems is low [15].

Blank liposomes were prepared to evaluate the baseline of the particle characteristics. The obtained sizes and polydispersity indexes of the liposomes are summarized in Table 2.

VANKA binds to liposome with a millimolar affinity. The larger the size of the liposomes, the stronger the binding of VANKA and liposomes. VANKA associates with liposome through its interaction with the head group of the lipids. The previous studies state that it is unlikely for the VANKA to penetrate deep into the lipids bilayer this means that VANKA does not interact and bind to lipid tails [37,38].

**Figure 2:**
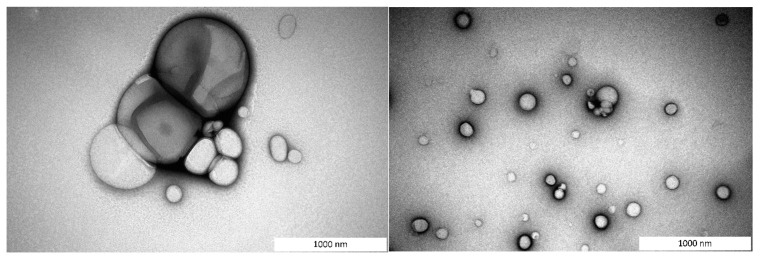
TEM pictures of VANKA loaded liposomes.

Liposomal sizes were verified by Transmission electron microscopy (TEM) (Figure 2). TEM analysis shows that prepared liposomes have a heterogeneous population in which it is possible to observe a close presence of two-layer structures. Also, Figure 2. shows that the majority of liposomes are spherical particles.

**Figure 3:**
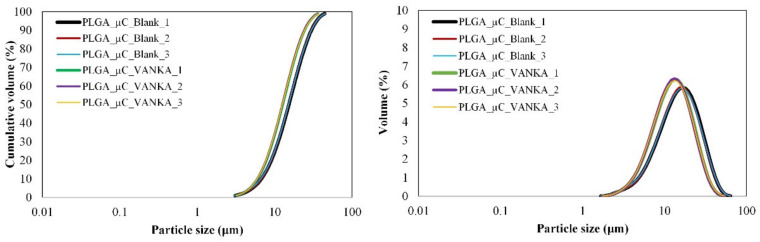
Particle size distribution of blank PLGA microcapsules and VANKA-loaded PLGA microcapsules.

The sizes of the microcapsules prepared in this study ranged from 3 µm to 46 µm (Figure 3). The mean size according to the granulometric analysis of blank PLGA microcapsules is 15.17 ± 0.11 µm, while the encapsulation of the VANKA resulted in a decrease in the particle size (mean size 12.60 ± 0.09 µm) (Table 3). According to the ANOVA test, the *p* values between the different groups range from 0.00001 to 0.00012. We assume that the decrease is observed due to the VANKA’s high solubility in water; it draws the water into the polymer membrane, thus accelerating the hydrolytic process [39].

**Figure 4:**
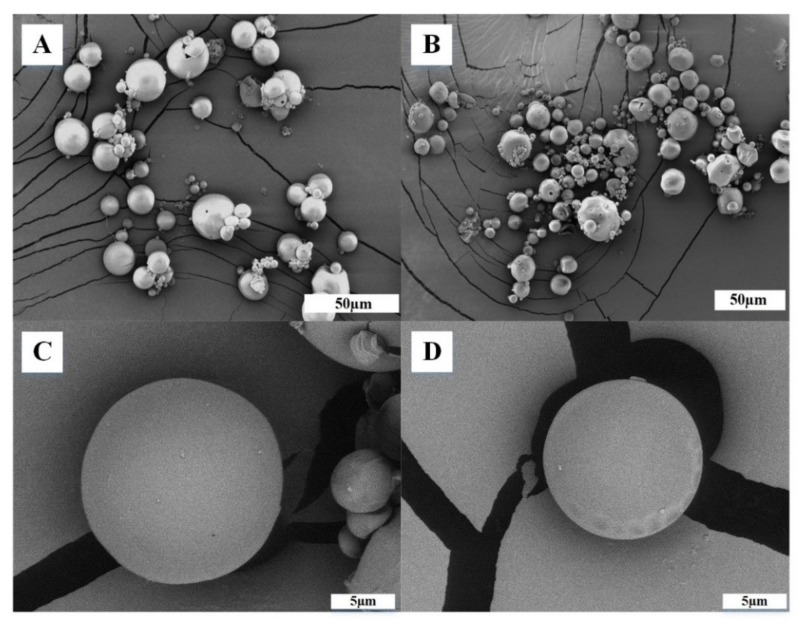
SEM pictures of surface of PLGA microcapsules (**A**,**C**) without and (**B**,**D**) with VANKA.

SEM images of the microcapsules with and without VANKA are shown in Figure 4. SEM photographs exhibited spherical particles with smooth surfaces, which indicated the absence of any drug crystal on the surface and confirmed the even distribution of the drug in the polymeric matrix (Figure 4B).

#### 3.1.2. Drug Load and Encapsulation Efficiency

The drug load and encapsulation efficiency are important parameters to evaluate the properties of microcapsules and liposomes (Table 4). The results of loading efficiency indicated that 56.44% of the initially used drug were encapsulated within liposomes and 12.30% were encapsulated within microcapsules. Their drug load resulted in 2.61 ± 0.01% for liposomes and 1.77 ± 0.03% for microcapsules. Although the results show that the loading of the drug into liposomes and microcapsules was very low, the MIC concentration can be reached by administering a certain amount of drug carriers to the PRF sample: 20 mg of liposomes and 50 mg of microcapsules, respectively. As the MIC value is negligible, it is not necessary to administer high concentrations of liposomes and microcapsules to the PRF. In the literature, the highest reported efficiency of drug encapsulation in liposomes is 35.58 ± 2.66%, drug load 2.55 ± 0.065% [15], which is lower than that achieved in our study, but in microcapsules, the drug encapsulation efficiency can reach 80 to 97% and drug load >54.6% when using PLGA 90:10, where the ratio of polymer to drug is superior [39]. A number of researchers have documented that highly hydrophilic drugs face the problems associated with low affinity for the polymer, resulting in unsatisfactory loading efficiency [40]. With a poor interaction between drug and polymer, the drug will tend to diffuse from the organic phase into the external aqueous environment during the spontaneous emulsification of the polymer. Although VANKA was completely dissolved in the organic phase, the drug could leak out during diffusion of the remaining dichloromethane from droplets of the organic phase into the aqueous dispersion medium [41]. The microcapsules used in this study with PLGA composition 50:50 have a faster (~20 days) degradation time, compared to PLGA 90:10 and PLGA 70:30 [29], and are more appropriate for the release of antibiotic agents with an optimal therapy time up to 14 days.

#### 3.1.3. Chemical Structure of VANKA Carriers

**Figure 5:**
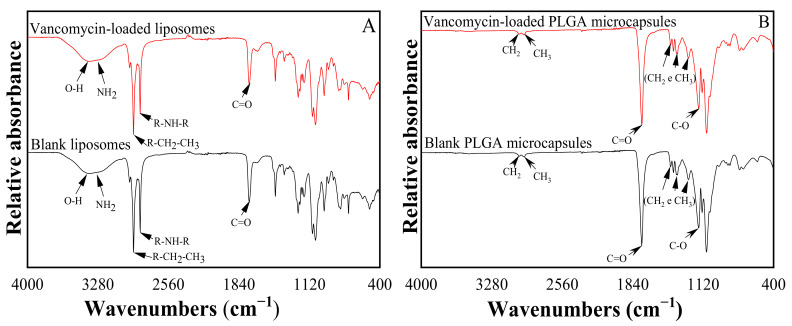
FTIR spectrum of (**A**) liposomes and (**B**) microcapsules.

The FTIR spectrum of liposomes is shown in Figure 5A. The VANKA molecule is characterized by oscillations of O-H and N-H_2_ bonds, the absorption observed is in the range of 3000 to 3600 cm^−1^, as well as fluctuations of C=O bonds, the absorption that occurs at 1670 cm^−1^.

The FTIR spectrum of the synthesized PLGA samples is shown in Figure 5B. In the PLGA microparticle sample spectrum, it is possible to observe a thick band in the range between 1750 cm^−1^ and 1740 cm^−1^, a characteristic range of carbonyl (C=O), present in the two monomers. Grouping band (C-O), between 1300 cm^−1^ and 1150 cm^−1^ can be observed, which is characteristic of ester groups. The absence of absorption bands between 3600 and 3400 cm^−1^ in Figure 5B, characteristic bands of the hydroxyl group, indicates that the PLGA copolymers are anhydrous.

No specific binding of VANKA with carrier materials, lipids (Figure 5A), or PLGA (Figure 5B) accordingly, were observed.

### 3.2. Characterization of Modified PRF Scaffold

**Figure 6:**
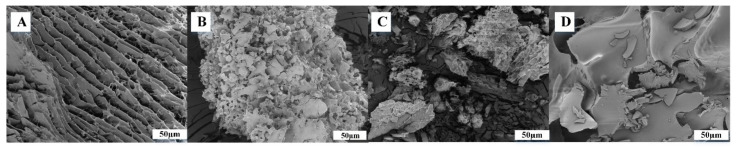
SEM pictures of PRF scaffolds: (**A**)–PRF; (**B**)—PRF/PLGA_μC_Blank; (**C**)—PRF/PLGA_μC_VANKA; (**D**)—PRF/VANKA.

**Figure 7:**
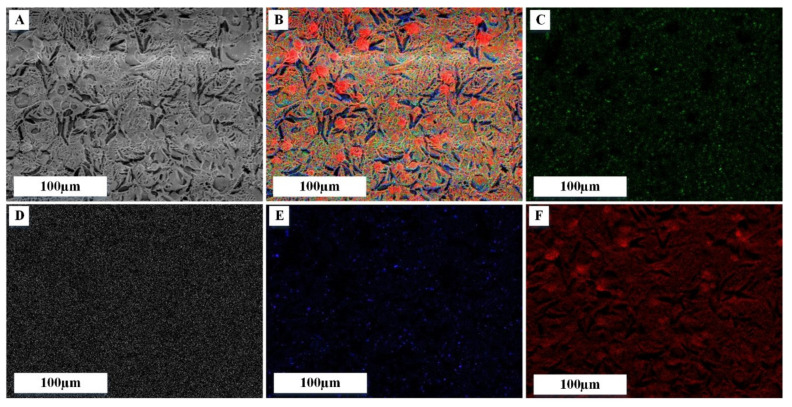
EDX mapping analysis on PRF/VANKA scaffold; (**A**) SEM image; (**B**) overall mapping elements on the same spot, corresponding to: (**C**) chloride, (**D**) potassium, (**E**) sodium and (**F**) oxygen mapping.

SEM images of PRF samples after lyophilization are shown in Figure 6. The surface morphology of PRF (Figure 6) shows a fibrous nature. PRF scaffolds without VANKA carriers and free drug show spaces between fibrin fibers that have been formed by thrombin (Figure 6A). In the literature, it has been found that greater the increase of thrombin concentration, the denser and thicker the fibers are [33]. All the prepared scaffolds contained fibrin networks and fibrin layers that interweave with the blank and VANKA-loaded microcapsules (Figure 6B,C). In turn, the PRF scaffolds with VANKA (Figure 6D) but without drug carriers exhibit a smooth surface, which indicates the absence of drug crystals on the surface. SEM-EDX analysis (see Figure 7) confirms the uniform distribution of the drug in the PRF scaffolds; the chloride distribution is attributed to the VANKA; the drug is in hydrochloride form.

**Figure 8:**
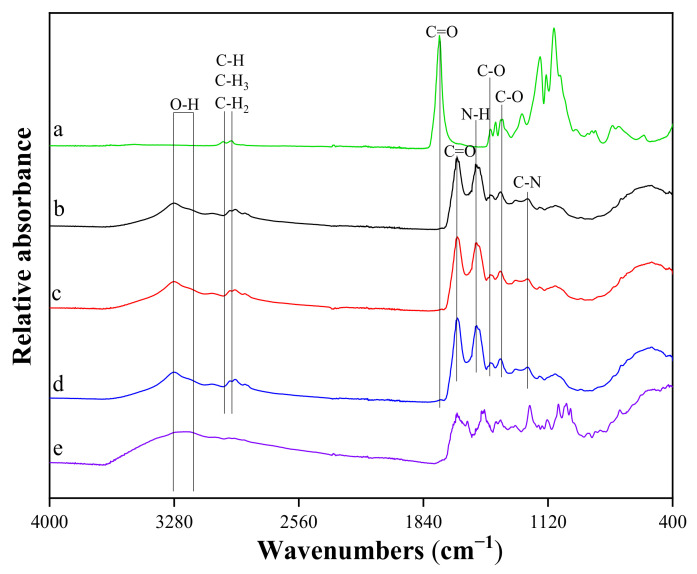
FTIR spectra of: (**a**) PLGA_ µC _Blank; (**b**) PRF scaffold; (**c**) PRF/ PLGA_ µC _Blank scaffold; (**d**) PRF/ PLGA_µC_VANKA scaffold; (**e**) VANKA.

The FTIR spectra of the samples (Figure 8) show the absorption bands characteristic of the fibrin phase in Figure 8: maximum at 1644 cm^−1^–amide I (C = O), maximum at 1531 cm^−1^–amide II (N-H), and maximum at 1259 cm^−1^–amide III (C-N). It can be seen that deformations of fibrin clots (either elongation or compression) have caused a marked rearrangement of the secondary structure of the proteins, which is reflected in the characteristic changes in the FTIR spectra. At the qualitative level, these changes could be described as a redistribution of the absorption intensity from higher to lower wavenumbers. This shift was confirmed by the differences in spectral peak positions observed in the different vibration modes of the peptides: amide I (decrease at 1649–1651 cm^−1^ and increase at 1620–1630 cm^−1^), amide II (decrease at 1540–1550 cm^−1^ and increase at 1530–1540 cm^−1^) and amide III (decrease at 1290–1320 cm^−1^ and increase at 1220–1240 cm^−1^). As found in the literature, the absorption of different proteins at higher wavenumbers (1649–1651 cm^−1^, 1540–1550 cm^−1^, and 1290–1320 cm^−1^) is mainly due to α-helical structures, whereas the lower wavenumbers (1620–1630 cm^−1^, 1530–1540 cm^−1^, and 1220–1240 cm^−1^) are mostly characteristic of β structures [42]. It can be concluded that the structure of β is more pronounced in the studied sample.

The FTIR analysis of PRF scaffolds with VANKA carriers showed a pronounced absorption maximum at 3304 cm^−1^, which indicates the presence of an OH group in the structure of the samples, which is characteristic of OH groups in the fibrin structure. In turn, the absence of this peak in the PLGA spectrum indicates that PLGA copolymers are anhydrous [43]. Absorption peaks at 1415 cm^−1^ (group C-O) and 1371 cm^−1^ (group C-O) correspond to the PLGA phase from the PLGA microcapsules added to the sample [44]. Absorption maxima at 2948 cm^−1^ and 2995 cm^−1^ are related to stretching vibrations of PLGA C-H, C-H_3_, and C-H_2_ functional groups [45].

The maximum at 1215 cm^−1^ indicates the presence of the hydroxyl group of the phenol in the structure of the samples, which is characteristic of the structure of VANKA [46]. From the FTIR spectra, it can be concluded that there is no specific binding of VANKA to PRF scaffold.

### 3.3. 3D Structure of Modified PRF Scaffolds

**Figure 9:**
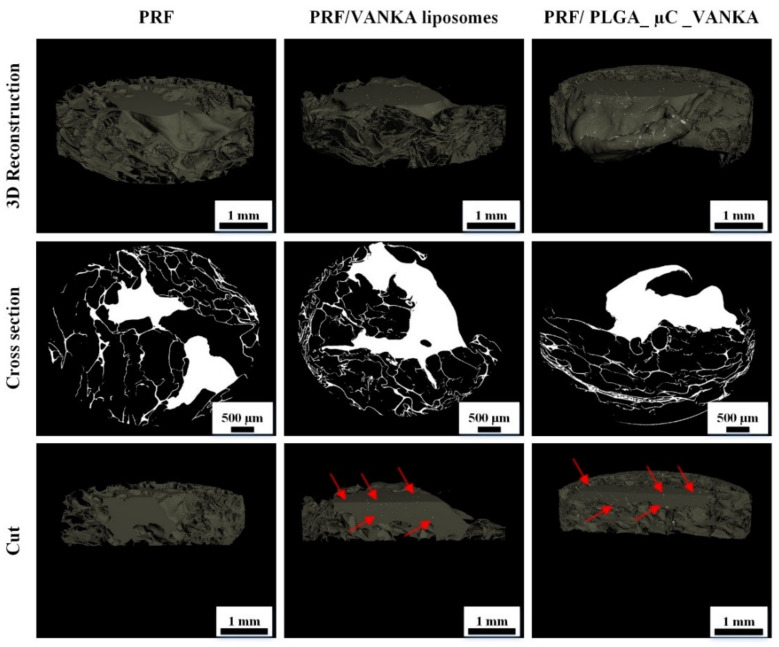
Micro-CT images of the PRF samples with VANKA liposomes (PRF/VANKA liposomes) and with VANKA microcapsules (PRF/PLGA_µC_VANKA) and the control samples without drug delivery systems (PRF); red arrows indicate the existence of delivery systems in the PRF samples.

**Figure 10:**
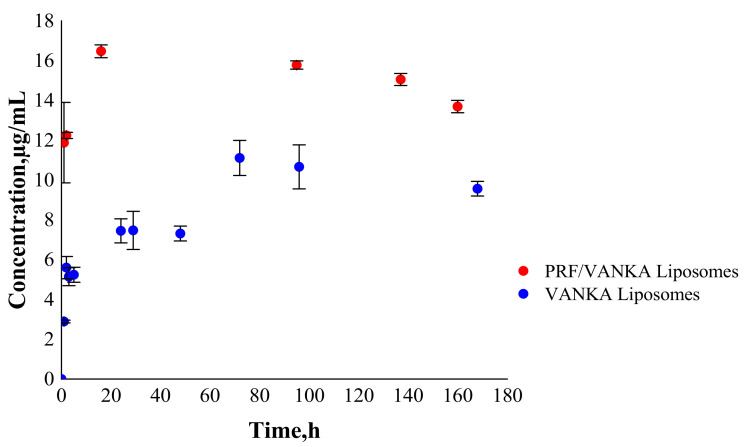
Drug release from PRF/VANKA liposomes and VANKA liposomes.

**Figure 11:**
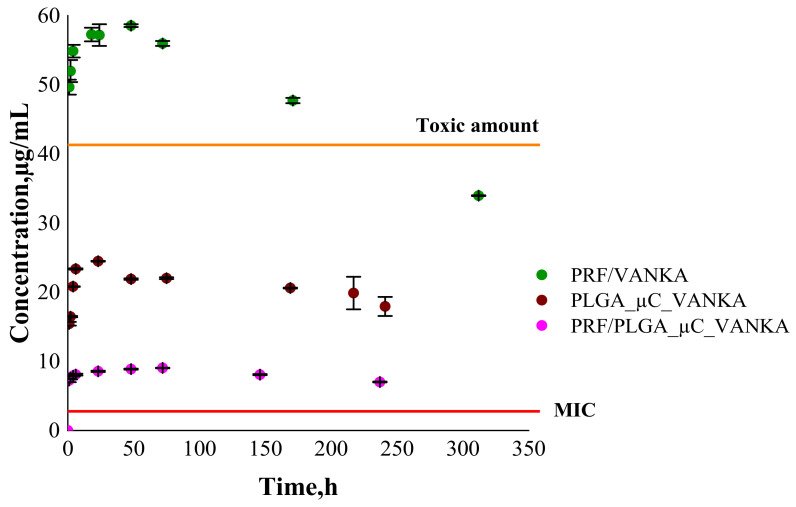
Drug release from PRF/VANKA scaffolds; PLGA_µC_VANKA and PRF/PLGA_µC_VANKA scaffolds.

Micro-CT images of three types of freshly made PRF samples after staining are shown in Figure 9. Micro-CT analysis of stained samples confirmed that VANKA carriers are encapsulated within the scaffolds during the preparation of the PRF scaffolds. In addition, the data shows the distribution of the medium according to the volume of the PRF framework. The upper portion seen in 3D micro-CT reconstruction images and cross-sectional images (colored white) is likely a PRF crust formed by coagulation of the sample. As seen in the images, VANKA liposomes are distributed directly in the PRF cortex. It is possible that a large amount of VANKA is released during the decomposition of the PRF crust. This is also supported by the release kinetics data (Figure 10). In turn, in the case of PRF/PLGA_µC_VANKA, it can be seen that the PLGA_µC_VANKA particles are scattered throughout the sample. Thus, as the sample degrades, only a portion of the encapsulated VANKA in the crust is rapidly released. The remainder of VANKA is released gradually and evenly (Figure 11).

### 3.4. VANKA Release Kinetics

Over time, VANKA degrades into CDP-1; thus, the concentration of the active form of VANKA decreases [20,21]. Consequently, an appropriate drug concentration quantification method has been developed. Therefore, instead of cumulative release, we indicate concentrations of free VANKA form at each measured time point.

The release kinetics of VANKA from PRF/VANKA liposomes is higher than from VANKA liposomes (Figure 10). The increased concentration of VANKA can possibly be explained by the fact that the Ca^2+^ ions in fibrin form a shell around the lipids, compressing them and thus destroying the liposomes [47,48]. Based on the results, it can be concluded that Ca^2+^ ions adversely affect the liposomes; thus, VANKA is released faster and in higher concentrations.

Based on the VANKA release studies from PLGA microcapsules (Figure 11), the kinetics of VANKA release from PRF/PLGA_µC_VANKA scaffolds are reduced fivefold compared to PRF/VANKA samples, ensuring controlled VANKA release and preventing burst release. These differences are also confirmed by the ANOVA test, which at *p* < 0.05 indicates a statistically significant difference. In contrast, comparing PLGA_μC_VANKA with PRF/PLGA_µC_VANKA scaffolds, it can be observed that the VANKA release concentration is reduced twofold. This suggests that the PRF scaffold inhibits the rapid release of VANKA. It was observed that with the release of VANKA from PRF /PLGA_µC_VANKA scaffolds, the highest VANKA concentrations are observed in the first hours, which is actually required to prevent possible postoperative infections. Results of VANKA release kinetics from PRF/VANKA samples exceeds the toxic amounts of VANKA during the first 24 h. As mentioned above, the therapeutic effect occurs at 20–40 µg/mL, but here, the concentration is above 49 µg/mL. It can be concluded that the introduction of VANKA in PRF scaffolds without VANKA carriers does not ensure a controlled delivery of the drug that is necessary for the therapeutic effect.

### 3.5. Antibacterial Properties of VANKA Containing PRF Scaffolds

PRF/VANKA liposome samples were not included in further experiments because, as demonstrated by the micro-CT images, liposomes cannot be homogeneously mixed into the PRF matrix and, as the PRF crust in which the liposomes are incorporated degrades more rapidly, rapid VANKA release is observed. The undesirable effect of Ca^2+^ ions on the structure of liposomes, which promotes the destruction of liposomes and results in faster VANKA release, cannot be excluded either.

**Figure 12:**
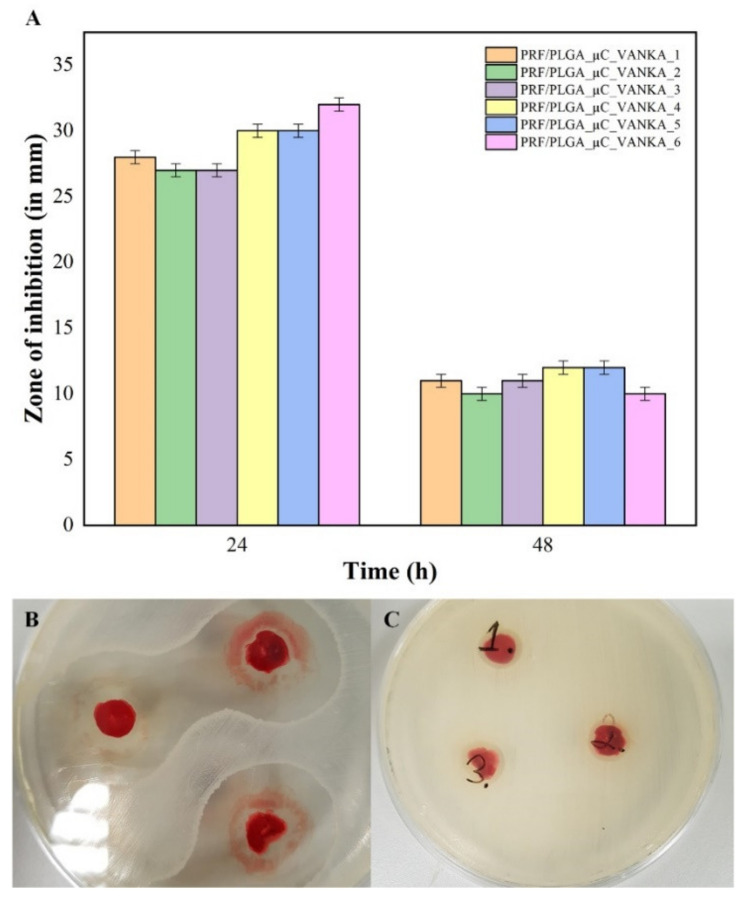
Antibacterial properties of PRF/PLGA_µC_VANKA samples: (**A**) inhibition zones in mm; (**B**) sterile areas around the PRF/PLGA_µC_VANKA samples after 24 h incubation; (**C**) sterile areas around the PRF/PLGA_µC_VANKA samples after 48 h incubation. The diameter of the petri dishes is 8.5 cm.

The maximum duration of antibacterial effect for VANKA-containing samples was observed for 48 h. For the first 24 h, the mean diameter of the sterile area around the samples is 30 mm. For the next 24 h, the diameter of the sterile area was reduced by 50% (Figure 12). No antibacterial properties were observed for samples without VANKA. The decrease of the sterile area after 48h can be explained by the experimental method, as the sample dries out during the incubation period and the diffusion of drugs from the sample is limited. To overcome this issue, we tested the antibacterial properties of the incubation medium of the sample rather that the sample itself (see Section 3.6).

### 3.6. Antibacterial Activity of Sample Incubation Medium

**Figure 13:**
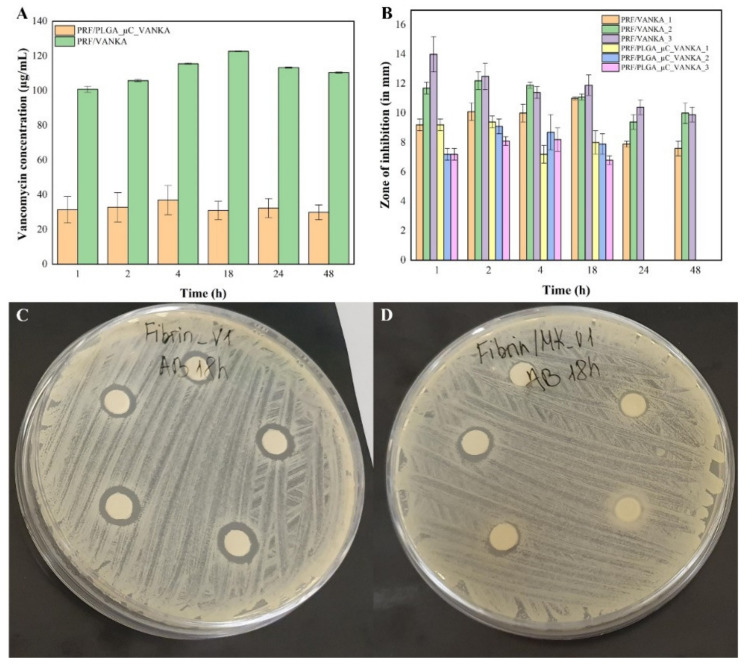
Antibacterial properties of sample incubation medium at different time points: (**A**) detected VANKA concentrations at sample incubation medium at different time points; (**B**) inhibition zones of disks impregnated with sample incubation medium at different time points; (**C**) sterile areas around the disks that were impregnated with PRF/VANKA sample incubation medium at 18-h time point; (**D**) sterile areas around the disks that were impregnated with PRF/PLGA_µC_VANKA sample incubation medium at 18-h time point. The diameter of the petri dishes is 8.5 cm.

The release of VANKA from the samples in the incubation medium was observed at all time points in the study (see Figure 13A). Concentrations of released VANKA from PRF/PLGA_µC_VANKA were 70% lower than PRF/VANKA samples. Due to the excretion of VANKA, antibacterial properties (sterile areas around the impregnated nitrocellulose disks) were not observed in PRF/PLGA_µC_VANKA samples at 24- and 48-h intervals. Significant differences in concentration between different types of samples gave proportionally higher antibacterial effects—the mean diameter of the sterile zone at 1-h intervals is 11 mm for PRF/VANKA incubation medium, but 8 mm for PRF/PLGA_µC_VANKA incubation medium. Differences in concentrations between samples of the same type were negligibly low and did not impact different antibacterial parameters, and the diameter of the sterile zones were maintained without significant differences.

## 4. Conclusions

According to the obtained results, we can conclude that the introduction of VANKA in PRF scaffolds without a carrier system does not ensure controlled delivery of active VANKA form at the therapeutic effect level and there is no specific binding of VANKA to PRF scaffold. This study confirms that the use of a carrier system can ensure controlled VANKA release for 6 to 10 days. A complete antibacterial effect lasts for 48 h, but with a rapid drop of effectiveness after the first 24 h. The methodology of antibacterial tests needs to be modified to ensure full VANKA release in the testing system.

Staining of the samples for micro-CT analysis is mandatory to distinguish the PRF scaffold from the encapsulated particles, thus showing the contrast between drug delivery systems and PRF. During 3D reconstruction of the samples, we observed that the liposomes are located at the upper layer of the sample, but the microcapsules are dispersed throughout the sample. These findings reinforce the observations of the VANKA release kinetics in this study and thus approve that micro-CT can be a tool for predicting the properties of drug delivery systems based on PRF.

## Figures and Tables

**Table 1:** Abbreviations of prepared samples.

Abbreviation	Sample
Blank liposomes	Liposomes without VANKA
VANKA liposomes	Liposomes with encapsulated VANKA
PLGA_µC_Blank	PLGA microcapsules without VANKA
PLGA_µC_VANKA	VANKA loaded PLGA microcapsules
PRF	Platelet-rich fibrin
PRF/VANKA liposomes	PRF with VANKA loaded liposomes
PRF/VANKA	PRF with VANKA- added as free drug powder, non-encapsulated
PRF/PLGA_µC_Blank	PRF without VANKA loaded PLGA microcapsules
PRF/PLGA_µC_VANKA	PRF with VANKA loaded PLGA microcapsules

**Table 2:** Size and polydispersity index of liposomes.

	Blank Liposomes	VANKA Liposomes
Average effective diameter, nm	1354.8 ± 100.6	932.7 ± 114.2
Polydispersity index	0.17	0.24

**Table 3:** Particle size distribution of VANKA-loaded PLGA microcapsules prepared via w/o/w technique.

Microcapsules	Particle Size ± SD, µm		
	d_10_	d_50_	d_90_
PLGA_µC_Blank	6.69 ± 0.08	15.17 ± 0.11	29.51 ± 0.44
PLGA_µC_VANKA	5.96 ± 0.03	12.60 ± 0.09	24.26 ± 0.18

**Table 4:** Drug loading and encapsulation efficiency of liposomes and microcapsules.

.	VANKA Liposomes	PLGA_µC_VANKA
Drug load, %	2.61 ± 0.01	1.77 ± 0.03
Encapsulation efficiency, %	56.44 ± 0.02	12.30 ± 0.05

## Data Availability

The data presented in this study are openly available in Zenodo at 10.5281/zenodo.5082110 (accessed on 7 July 2021).
